# Reduced Ebola vaccine responses in CMV^+^ young adults is associated with expansion of CD57^+^KLRG1^+^ T cells

**DOI:** 10.1084/jem.20200004

**Published:** 2020-05-15

**Authors:** Georgina Bowyer, Hannah Sharpe, Navin Venkatraman, Pierre Birahim Ndiaye, Djibril Wade, Nicole Brenner, Alex Mentzer, Catherine Mair, Tim Waterboer, Teresa Lambe, Tandakha Dieye, Souleymane Mboup, Adrian V.S. Hill, Katie J. Ewer

**Affiliations:** 1 The Jenner Institute, University of Oxford, Oxford, UK; 2 Centre Hospitalier Universitaire le Dantec, Dakar, Senegal; 3 Infections and Cancer Epidemiology, German Cancer Research Center, Heidelberg, Germany

## Abstract

CMV is associated with immunosenescence and reduced vaccine responses in the elderly (>70 yr). However, the impact of CMV in young adults is less clear. In this study, healthy UK and Senegalese adults aged 18–50 yr (average, 29 yr) were vaccinated with the Ebola vaccine candidate chimpanzee adenovirus type 3–vectored Ebola Zaire vaccine (ChAd3-EBO-Z) and boosted with modified vaccinia Ankara Ebola Zaire–vectored (MVA–EBO-Z) vaccine. CMV carriage was associated with an expansion of phenotypically senescent CD4^+^ and CD8^+^ T cells expressing CD57 and killer cell lectin-like receptor G1 (KLRG1), which was negatively associated with vaccine responses in both cohorts. Ebola-specific T cell responses induced by vaccination also contained significantly increased frequencies of terminally differentiated CD57^+^KLRG1^+^ cells in CMV seropositive (CMV^+^) individuals. This study suggests that CMV can also affect vaccine responses in younger adults and may have a particularly marked impact in many developing countries where CMV seroprevalence is almost universal.

## Teaser

CMV is emerging as a key driver of immunosenescence. Bowyer et al. report that an expansion of phenotypically senescent CD4^+^ and CD8^+^ T cells is associated with reduced responses to Ebola vaccines ChAd3–EBO-Z and MVA–EBO-Z in young UK and Senegalese adults.

## Graphical Abstract

**Figure:**
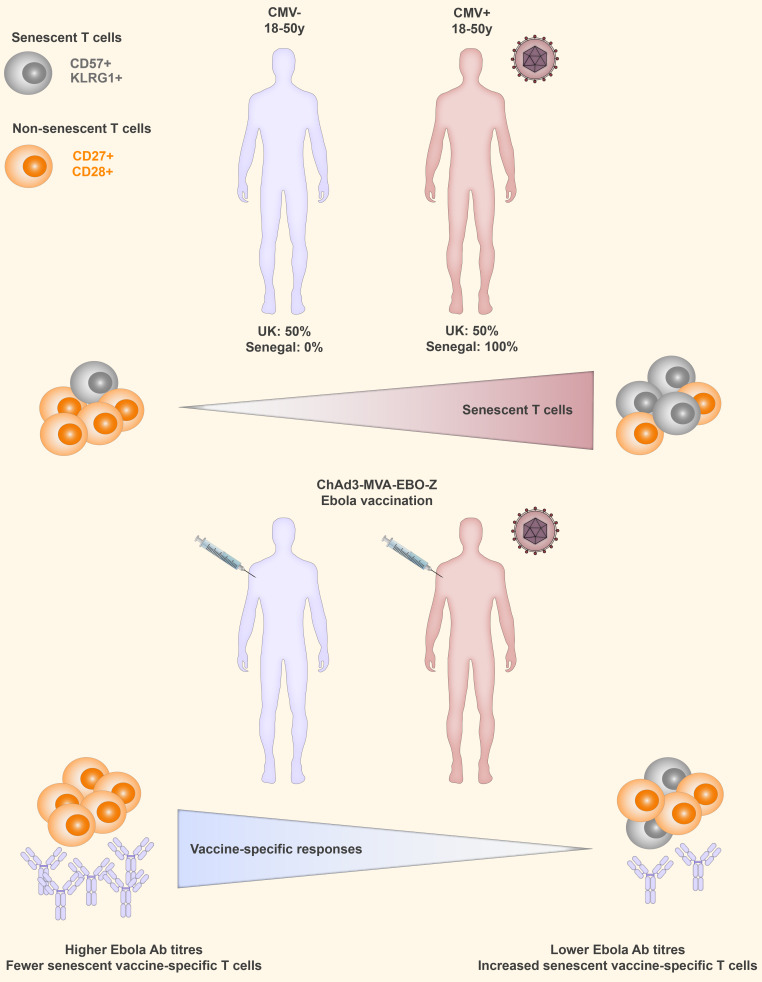

## Introduction

Human CMV is a highly prevalent β-herpes virus that establishes life-long latent infections. Around 40%–60% of young adults in developed countries are infected (Zuhair et al., 2019), increasing to >90% in elderly adults (Staras et al., 2006). CMV seroprevalence in developing countries is often higher, with 80%–90% of young adults seropositive (Zuhair et al., 2019). There is increasing evidence that CMV plays a significant role in immunosenescence and is characterized by a gradual accumulation of highly differentiated effector memory T cells in a process known as “memory inflation” (Karrer et al., 2003; Sylwester et al., 2005; O’Hara et al., 2012; Hosie et al., 2017). Although inflationary T cells do not express classical exhaustion markers such as programmed cell death protein 1 (PD-1), they typically lose expression of costimulatory receptors CD27 and CD28 and gain expression of the inhibitory receptor killer cell lectin-like receptor G1 (KLRG1) and the terminal differentiation marker CD57 (Henson et al., 2012; Klenerman and Oxenius, 2016). Functionally, these cells have reduced proliferative capacity, increased activation of senescence signaling pathways, and a greater susceptibility to apoptosis in vitro (Henson et al., 2012).

In elderly populations, these CMV-driven immune changes have been associated with reduced vaccine responses and an increased risk of mortality (Wikby et al., 1994, 2002; Ferguson et al., 1995; Trzonkowski et al., 2003; Moro-García et al., 2012; Derhovanessian et al., 2013, 2014). However, although marked changes in immune phenotype and significant proportions of CMV-specific T cells are also observed in healthy younger seropositive adults and children (Turner et al., 2014; Brodin et al., 2015; van den Heuvel et al., 2016), the impact on responses to vaccination or infection is less clear, and most studies have been conducted in populations within developed countries (Sidorchuk et al., 2004; Holder et al., 2010; Saghafian-Hedengren et al., 2013; Turner et al., 2014; Furman et al., 2015; van den Berg et al., 2018).

Reduced vaccine responses are frequently observed in developing countries, with an increased burden of pathogen exposure thought to be one driving factor (Lagos et al., 1999; Qadri et al., 2003; Serazin et al., 2010; Lopman et al., 2012). However, direct evidence of an association between pathogen exposure, altered immune phenotypes, and reduced vaccine responses is lacking. During the 2014–2016 Ebola outbreak in West Africa, we conducted two Phase I clinical trials of the Ebola vaccine candidates chimpanzee adenovirus serotype 3 (ChAd3) and modified vaccinia virus Ankara (MVA), both expressing Zaire Ebola glycoprotein (EBO-Z; Venkatraman et al., 2018). The trials were run concurrently in Oxford, UK, and Dakar, Senegal, with healthy UK adults aged 18–50 yr (*n* = 16; average, 33 yr) and Senegalese adults aged 18–50 yr (*n* = 40; average, 28 yr) in the matched dose groups receiving the same vaccine regimen: 3.6 × 10^10^ viral particles of ChAd3–EBO-Z at day 0, boosted with 1 × 10^8^ plaque-forming units of MVA–EBO-Z 1 wk later. This trial design provided a rare opportunity for direct comparison of vaccine immunogenicity in populations within a developed country and a developing country. We discovered a novel association between CMV-associated changes to the T cell repertoire and a reduction in Ebola vaccine responses in healthy young UK and Senegalese adults.

## Results and discussion

### CMV seropositivity is associated with reduced responses to ChAd3-MVA–EBO-Z vaccination

**Figure 1.**
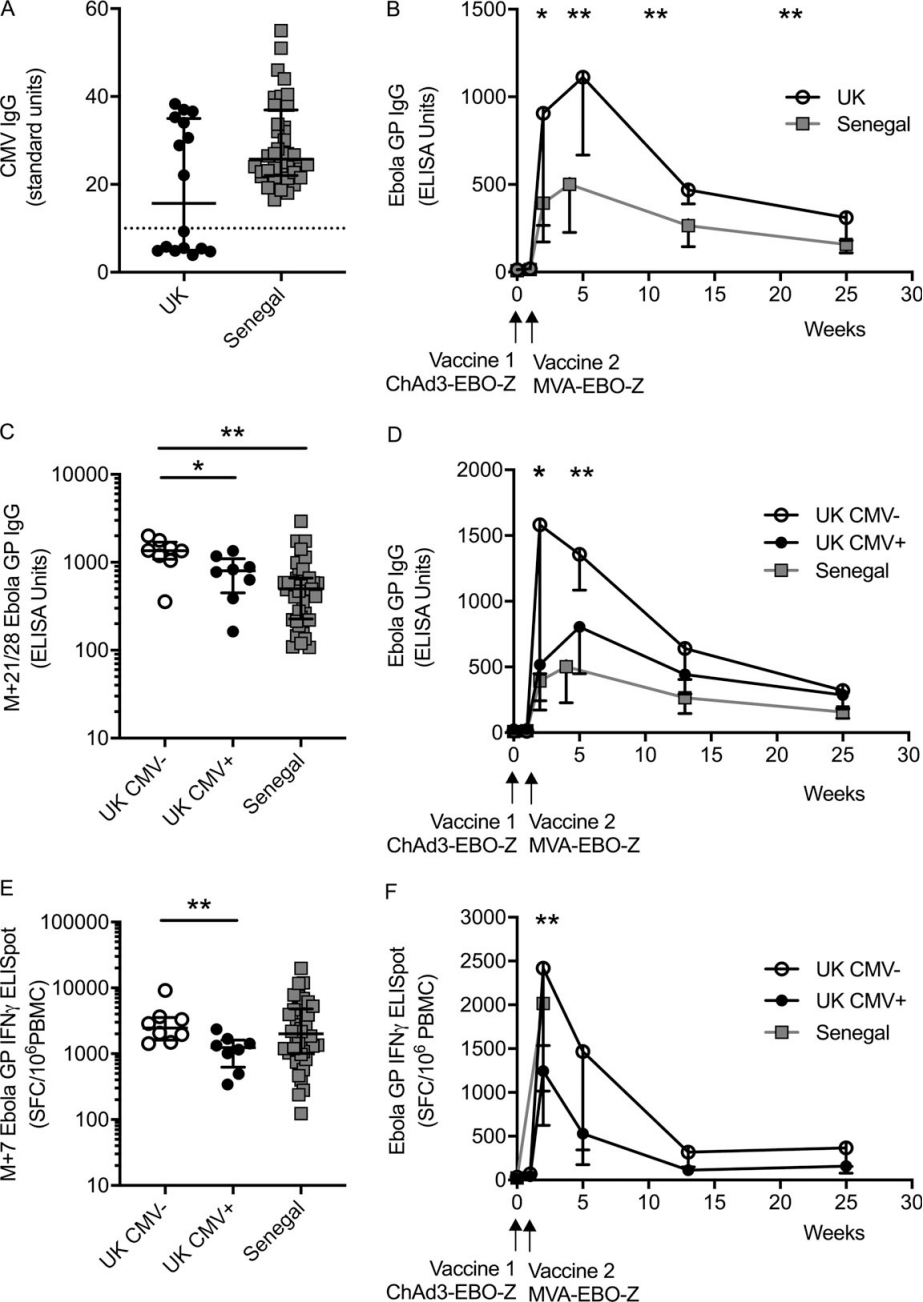
**Vaccine responses are reduced in CMV^+^ young adults.** **(A)** CMV IgG titers measured by ELISA in each cohort (UK, *n* = 16; Senegal, *n* = 40). Dashed line indicates seropositive threshold. **(B)** Time courses of Ebola-specific antibody responses after vaccination in both cohorts. Median and IQRs shown. Mann–Whitney analyses between cohorts at each time point (UK, *n* = 16; Senegal, *n* = 40). **(C)** Peak vaccine-specific antibody responses (3–4 wk after MVA vaccination, M+21/28). Kruskal–Wallis with Dunn’s post-test analysis across all three groups, P = 0.0034 (UK CMV^−^, *n* = 8; UK CMV^+^, *n* = 8; Senegal, *n* = 40). **(D)** Time courses of vaccine-specific antibody responses stratified by CMV serostatus (medians and IQRs; UK CMV^−^, *n* = 8; UK CMV^+^, *n* = 8; Senegal, *n* = 40). **(E)** IFN-γ ELISPOT responses at M+7. Kruskal–Wallis with Dunn’s post-test comparison across groups across all three groups, P = 0.096. CMV^−^ (UK CMV^−^, *n* = 8; UK CMV^+^, *n* = 8; Senegal, *n* = 40). Medians shown for column graphs. Error bars indicate IQRs. SFC/10^6^ PBMCs = spot-forming cells per million PBMCs. **(F)** Time courses of T cell responses measured by IFN-γ ELISPOT. Median and IQRs of responses against summed Ebola GP peptide pools. Only baseline (D0) and peak (D14, M+7) were measured in the Senegalese cohort. (UK CMV^−^ *n* = 8, UK CMV^+^ *n* = 8, Senegal *n* = 40). *, P < 0.05; **, P < 0.01.

Of the UK cohort, 50% (8/16) of participants were positive for CMV IgG, while 100% (40/40) of the Senegalese cohort was positive (Fig. 1 A), which is in line with previous reports in these populations (Cannon et al., 2010; Adland et al., 2015). Titers of CMV IgG were comparable in UK CMV^+^ and Senegalese participants. Ages of participants in the UK CMV^−^, UK CMV^+^, and Senegalese cohorts were comparable and did not correlate with CMV IgG titer (Table S1). Demographics of both cohorts are summarized in Table S1.

As reported in the primary clinical trial results (Venkatraman et al., 2018), vaccine-specific antibody responses were significantly lower in the Senegalese cohort than in the UK cohort at peak and late time points (Fig. 1 B). However, when stratified by CMV serostatus, vaccine-specific antibody responses were significantly lower in CMV^+^ than CMV^−^ UK participants (Fig. 1, C and D; P = 0.028). Senegalese participants, who were all CMV^+^, had vaccine-specific antibody responses that were comparable to those of UK CMV^+^ participants (P = 0.52) but significantly lower than those of CMV^−^ participants (P = 0.0032).

CMV carriage was also associated with a significant reduction in vaccine-specific T cell responses (measured by IFN-γ ELISPOT) in the UK cohort (Fig. 1, E and F; P = 0.007). However, there was no significant difference in vaccine-specific T cell responses between either the UK CMV^−^ or the CMV^+^ group and the Senegalese cohort.

Although a range of studies have shown contradictory findings on the impact of CMV on immune responses, with some demonstrating a negative effect (Trzonkowski et al., 2003; Derhovanessian et al., 2013; Turner et al., 2014; Frasca et al., 2015; Wagner et al., 2018), some a positive effect (Miles et al., 2008; Holder et al., 2010; Wald et al., 2013; Furman et al., 2015), and others no effect (den Elzen et al., 2011; O’Connor et al., 2014), it is likely that a combination of factors contributes to these differing results. First, the impact of CMV on heterologous immune responses may differ between primary and memory responses. The majority of studies focused on boosting memory responses, while we examined responses to the neoantigen Ebola glycoprotein (GP) in naive individuals.

Second, there is likely an effect of age (or length of CMV carriage). In contrast to studies in older adults, in which CMV has often been negatively associated with immune responses (Derhovanessian et al., 2013, 2014), various studies in children and infants have demonstrated no effect or positive effects of CMV carriage (Miles et al., 2008; Holder et al., 2010; van den Heuvel et al., 2016), although CMV has also been associated with an increased risk of tuberculosis disease in infants (Müller et al., 2019). In particular, the expansion of terminally differentiated CD57^+^CD27^−^CD28^−^CD4^+^ T cells in CMV^+^ adults is not always apparent in CMV^+^ infants, even when CD57^−^CD27^−^CD28^−^CD4^+^ T cells are expanded (Miles et al., 2008). These cells are thought to accumulate with repeated antigen exposure and therefore expand over time in CMV^+^ individuals (Pourgheysari et al., 2007). These cells may play an active role in immunosuppression in an antigen-independent manner, as demonstrated recently (Tovar-Salazar and Weinberg, 2017). Therefore, we assessed the T cell populations in our cohorts to determine if these cells were associated with the reduction in vaccine responses in CMV^+^ individuals.

### CMV^+^ young adults have marked differences in global T cell repertoire

**Figure S1.**
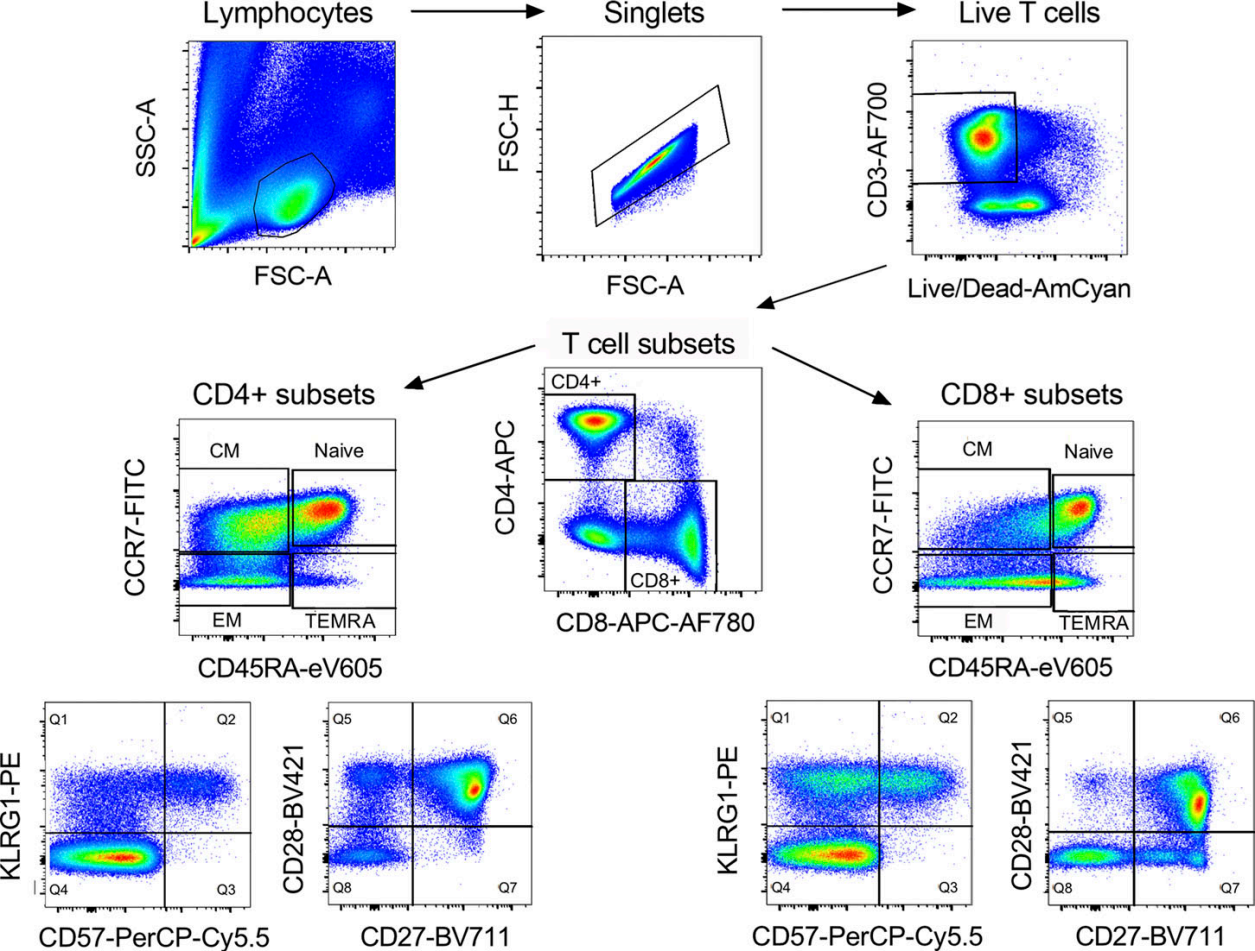
**Gating strategy for memory T cell phenotyping panel.** CD4^+^ and CD8^+^ T cells were gated in the order shown. Gates for different memory populations based on expression of CCR7, CD45RA, KLRG1, CD57, CD27, or CD28 as shown were then applied to each of the subsets depending on the analysis being conducted. EM, effector memory; FSC-A, forward scatter area; FSC-H, forward scatter height; SSC-A, side scatter area.

We assessed frequencies of CD4^+^ and CD8^+^ T cells and memory populations defined as: CD45RA^+^CCR7^+^ naive, CD45RA^−^CCR7^+^ central memory, CD45RA^−^CCR7^−^ effector memory, and CD45RA^+^CCR7^−^ terminal effector memory reexpressing CD45RA (TEMRA; Mahnke et al., 2013; Fig. S1).

**Figure S2.**
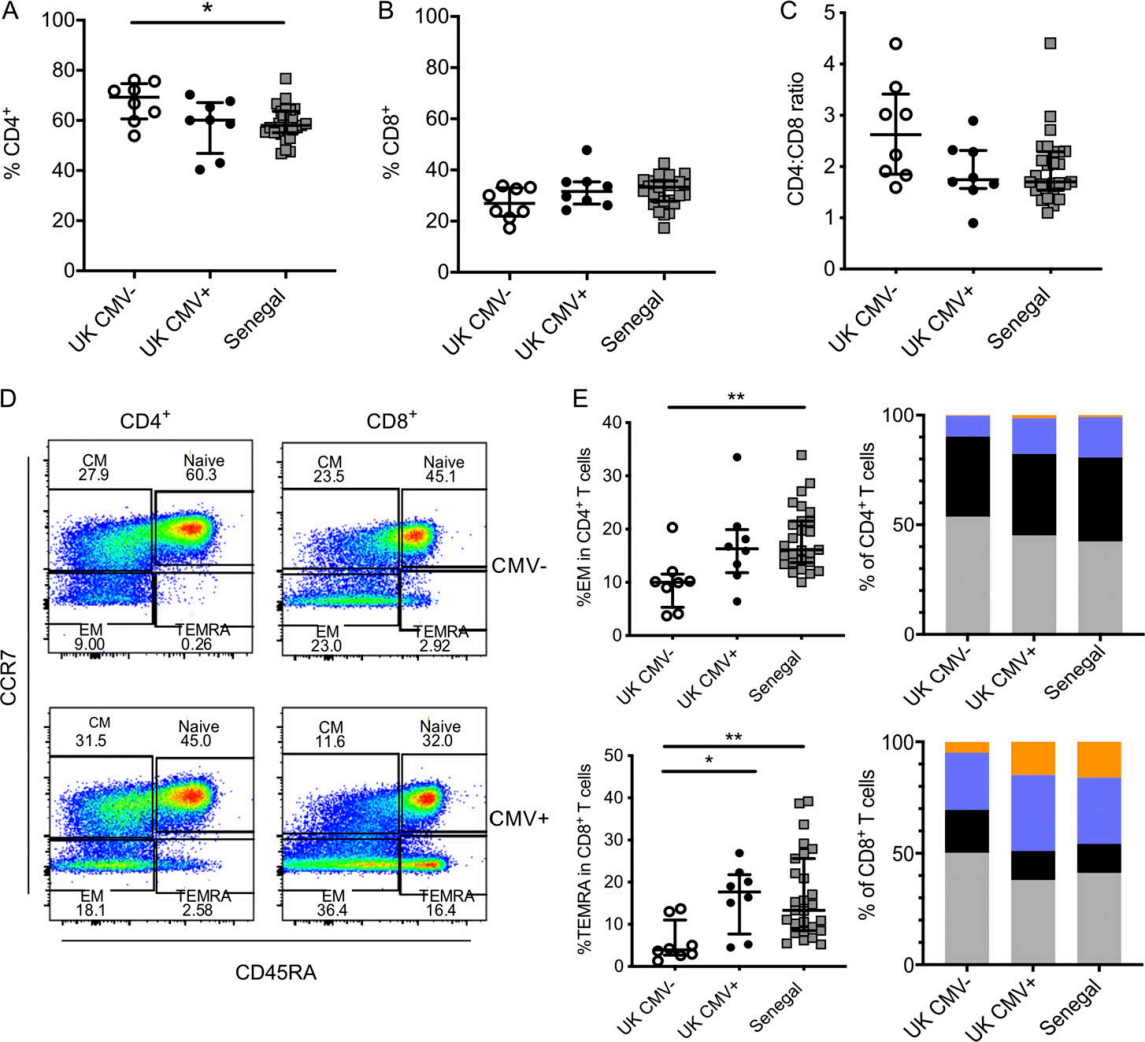
**Altered CD4^+^ and CD8^+^ T cell populations in CMV^+^ young adults.** **(A)** Frequency of CD4^+^ T cells in UK CMV^−^ and CMV^+^ individuals and in Senegalese individuals (all Senegalese individuals were CMV^+^). **(B and C)** Frequency of CD8^+^ T cells (B) and ratio of CD4:CD8 T cells (C). **(D)** Representative flow cytometry plots of subsets within CD4^+^ and CD8^+^ T cells in CMV^−^ and CMV^+^ individuals; naive: CD45RA^+^CCR7^+^, central memory (CM): CD45RA^−^CCR7^+^, effector memory (EM): CD45RA^−^CCR7^−^, and TEMRA: CD45RA^+^CCR7^−^. **(E)** Subsets within CD4^+^ and CD8^+^ T cells as defined by the gating strategy above. Clockwise from top left: Proportion of EMs in CD4^+^ T cells, geomeans of subset frequencies within CD4^+^ T cells, geomeans of subset frequencies within CD8^+^ T cells, and proportion of TEMRA in CD8^+^ T cells. Orange (top bar), TEMRA; blue (second from top), effector memory; black (third from top), CM; gray (bottom bar), naive. Kruskal–Wallis analysis with Dunn’s post-test comparisons of groups. Medians and IQRs shown. *, P < 0.05; **, P < 0.01.

CMV has been linked to a decreased CD4:CD8 ratio and an associated reduction in responses to novel antigens in elderly populations (Klenerman and Oxenius 2016; Wagner et al., 2018). This reversal of CD4:CD8 ratio appears to be predominantly driven by the expansion of CD8^+^ T cells specific for CMV (Hadrup et al., 2006). However, in the younger adults in our study, CMV seropositivity was associated with a reduced frequency of CD4^+^ T cells, while there was no significant difference in CD8^+^ T cells (Fig. S2, A and B). This resulted in a number of CMV^+^ participants with low or inverted CD4:CD8 ratios (Fig. S2 C). Additionally, CMV^+^ participants had increased proportions of effector memory and TEMRA CD4^+^ and CD8^+^ T cells (Fig. S2, D and E).

CMV-specific CD4^+^ T cells are thought to play an important role in containing CMV infection, and in mouse models, CD4^+^ T cells were abundant in infected peripheral tissues (Reuter et al., 2005; Verma et al., 2015). A reduction in the CD4:CD8 ratio in healthy young CMV^+^ adults, which has also been reported in other studies (Turner et al., 2014), could be caused by a reduction in circulating CD4^+^ T cells as they are recruited to peripheral sites of infection. As infected individuals age, multiple reactivation events over many decades cause a gradual expansion of CMV-specific CD8^+^ T cells, which then become the prominent factor driving the CD4:CD8 ratio down (Hadrup et al., 2006).

### Memory T cells in CMV^+^ young adults are phenotypically senescent

Lifelong CMV infection is associated with a gradual expansion of T cells that have down-regulated classic costimulatory receptors (CD27, CD28) and up-regulated inhibitory receptors, such as KLRG1, and markers of terminal differentiation, such as CD57 (Klenerman, 2018). As the expansion of these cells has been associated with immunosenescence and reduced survival in CMV^+^ elderly adults (Olsson et al., 2000; Pourgheysari et al., 2007), we assessed the proportions of T cells expressing these markers at baseline in the younger adults in this study.

**Figure 2.**
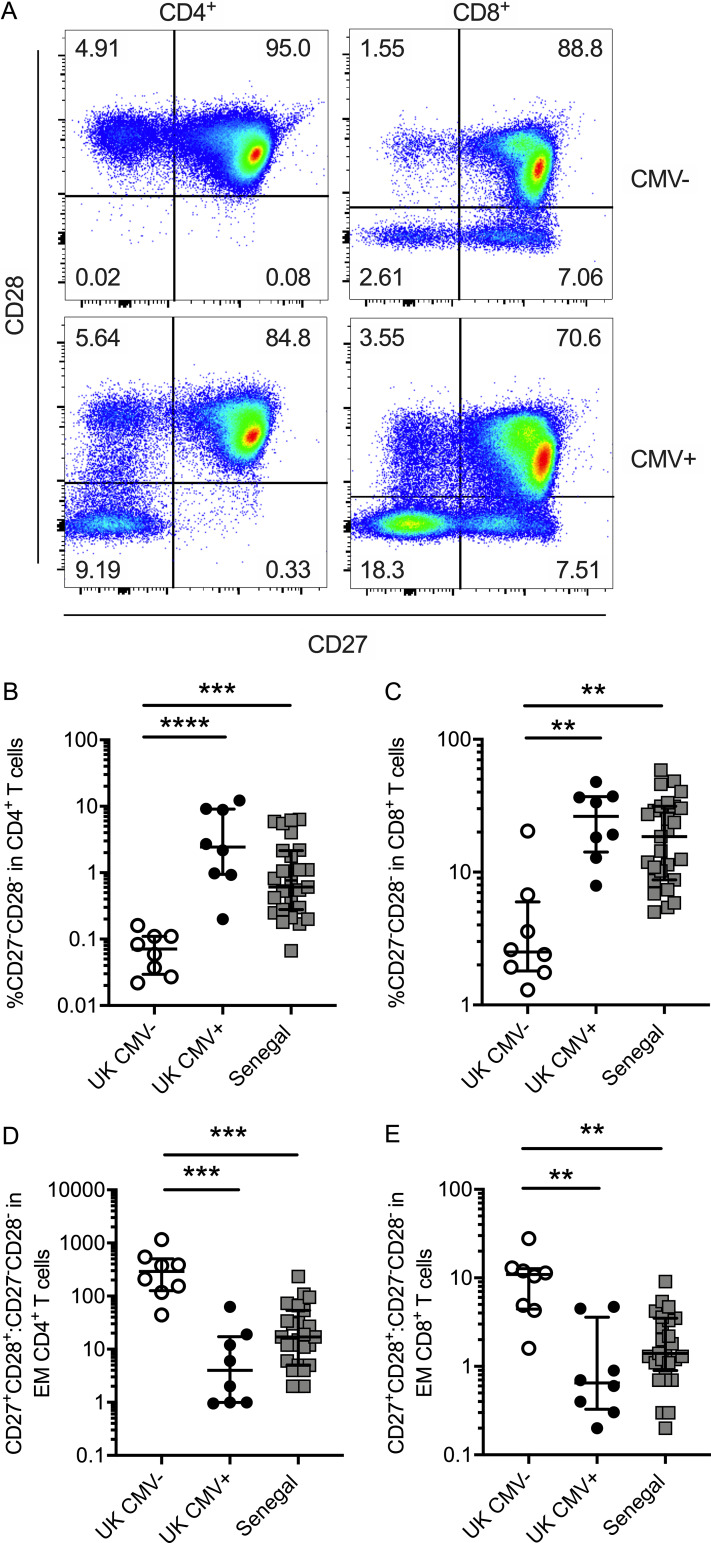
**CD27 and CD28 expression is reduced on T cells from CMV^+^ young adults.** **(A)** Representative flow plots showing CD27 and CD28 expression on CD4^+^ (left) and CD8^+^ (right) T cells in CMV^−^ (top row) and CMV^+^ (bottom row) individuals. **(B)** Frequency of CD4^+^ T cells that are CD27^−^CD28^−^. **(C)** Frequency of CD8^+^ T cells that are CD27^−^CD28^−^. **(D and E)** Ratio of CD27^+^CD28^+^ to CD27^−^CD28^−^ within effector memory (EM; CD45RA^−^CCR7^−^) CD4^+^ (D) and effector memory CD8^+^ (E) T cells. UK CMV^−^, *n* = 8; UK CMV^+^, *n* = 8; Senegal, *n* = 27. Kruskal–Wallis analyses with Dunn’s post-test across all three groups. **, P < 0.01; ***, P < 0.001; ****, P < 0.0001. Medians shown for all column graphs. Error bars indicate IQRs.

Both CMV^+^ UK and Senegalese participants had significantly increased frequencies (up to 10-fold higher) of total CD4^+^ and CD8^+^ T cells lacking expression of CD27 and CD28 compared with CMV^−^ participants (Fig. 2, A–C). In some individuals, over 10% of CD4^+^ T cells and 50% of CD8^+^ T cells did not express either CD27 or CD28. The frequency of CD27^−^CD28^−^ T cells was also increased within effector memory T cell populations in CMV^+^ individuals (Fig. 2, D and E).

**Figure 3.**
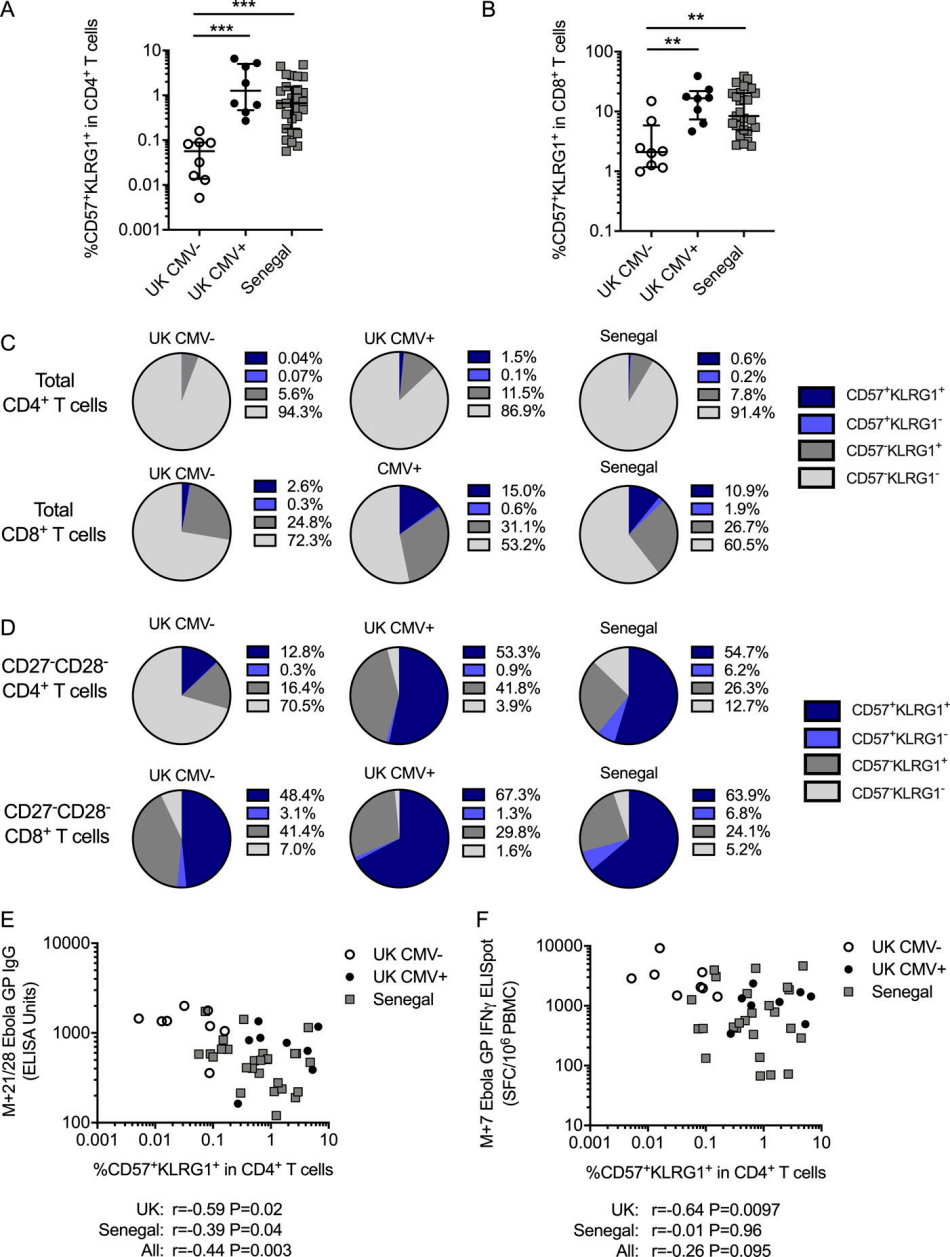
**CD57^+^KLRG1^+^ T cells are expanded in CMV^+^ young adults and negatively correlate with vaccine responses.** **(A and B)** Frequency of CD57^+^KLRG1^+^ double-positive cells within total CD4^+^ T cells (A) and total CD8^+^ T cells (B). Kruskal–Wallis analyses with Dunn’s post-comparison tests. **, P < 0.01; ***, P < 0.001. Medians shown for column graphs. Error bars indicate IQRs. **(C)** Geomean subsets based on the expression of CD57 and KLRG1 in total CD4^+^ (top row) or CD8^+^ (bottom row) T cells in each group. **(D)** Geomean subsets based on the expression of CD57 and KLRG1 in CD27^−^CD28^−^CD4^+^ (top row) or CD27^−^CD28^−^CD8^+^ (bottom row) T cells in each group. **(E and F)** Relationship between the proportion of CD57^+^KLRG1^+^ double-positives within CD4^+^ T cells and peak antibody responses measured by ELISA (E) and peak T cell responses against Ebola GP measured by IFN-γ ELISPOT (F). Spearman’s rank analyses. UK CMV^−^ *n* = 8, UK CMV^+^ *n* = 8, Senegal *n* = 27. SFC, spot-forming cell.

CMV^+^ participants also had significantly increased frequencies of CD4^+^ and CD8^+^ T cells expressing both the terminal differentiation marker CD57 and the inhibitory receptor KLRG1 (Fig. 3, A and B; P = 0.0003 and P = 0.0029, respectively), which may further mark cells that have undergone a large number of divisions, have low proliferative potential, express senescence markers, and have reduced cytokine production capacity (Ibegbu et al., 2005; Koch et al., 2008; Strioga et al., 2011). Although the majority of CD4^+^ T cells were CD57^−^KLRG1^−^, both CD57^−^KLRG1^+^ and CD57^+^KLRG1^+^ were expanded in CMV^+^ compared with CMV^−^ participants (Fig. 3 C). Similarly, these populations were also expanded within CD8^+^ T cells in the CMV^+^ individuals (over 10% CD57^+^KLRG1^+^ in CMV^+^ compared with just 2.6% in CMV^−^ individuals, although two individuals had an expansion of these cells despite being CMV^−^).

The frequency of CD57^+^KLRG1^+^ cells was also increased within CD27^−^CD28^−^CD4^+^ and CD27^−^CD28^−^CD8^+^ T cell subsets in CMV^+^ participants (Fig. 3 D). While the majority (>70%) of CD27^−^CD28^−^CD4^+^ T cells were CD57^−^KLRG1^−^ in CMV^−^ participants, over half of this subset expressed CD57 and KLRG1 in CMV^+^ participants. Similarly, CD57 and KLRG1 expression was increased in CD27^−^CD28^−^ CD8^+^ T cells in CMV^+^ individuals. These findings demonstrate that there is an expansion of highly differentiated CD4^+^ and CD8^+^ T cells expressing markers of senescence even in young CMV^+^ adults.

### Increased proportions of CD57^+^KLRG1^+^ CD4^+^ T cells are associated with reduced vaccine responses in CMV^+^ young adults

Expansions of such highly differentiated memory T cells have been associated with reduced vaccine responses in the elderly (Goronzy et al., 2001; Saurwein-Teissl et al., 2002; Derhovanessian et al., 2013, 2014), possibly by restricting the “immunological space” and reducing the production of naive T cells, thereby reducing responses to novel antigens (Franceschi et al., 2000). In our study, the expansion of terminally differentiated CD57^+^KLRG1^+^ CD4^+^ T cells in CMV^+^ young adults before MVA–EBO-Z vaccination was negatively associated with vaccine-specific antibody responses (Ebola GP–specific IgG at M+28) in both the UK and Senegalese cohorts (Fig. 3 E). The frequency of these cells was also negatively associated with vaccine-specific T cell responses (peak IFN-γ ELISPOT) in the UK cohort, but not in the Senegalese cohort (Fig. 3 F).

Chronic antigen stimulation in persistent viral infections can drive T cell exhaustion in addition to T cell senescence. These processes are distinct and are characterized by different sets of markers (Akbar and Henson, 2011). While exhausted T cells have a reduced proliferative potential, decreased cytotoxicity, and impaired cytokine secretion (Wherry and Kurachi, 2015), senescent T cells are terminally differentiated with limited proliferative capacity but retain some (altered) functionality (Strioga et al., 2011). CD57 and KLRG1 are commonly used markers of senescent T cells, while exhausted T cells generally express these at low levels (Larbi and Fulop, 2014). Although T cells expanded in CMV^+^ individuals have been shown to have low proliferative capacity and express senescence markers such as KLRG1 and CD57 (Vieira Braga et al., 2015), they are not exhausted as they are still highly cytotoxic and produce Th1 cytokines in response to sporadic viral reactivation (Klenerman and Oxenius, 2016). Although the expanded T cells in our cohorts express markers traditionally associated with senescence, it is unclear what the exact functional state of this subset is. Investigating the transcriptional profile of these cells could provide insights into the potential mechanisms underlying the association with reduced vaccine responses and would be a priority for future studies.

### CMV^+^ young adults produce vaccine-specific T cell responses with an increased proportion of terminally differentiated CD57^+^KLRG1^+^ cells

**Figure 4.**
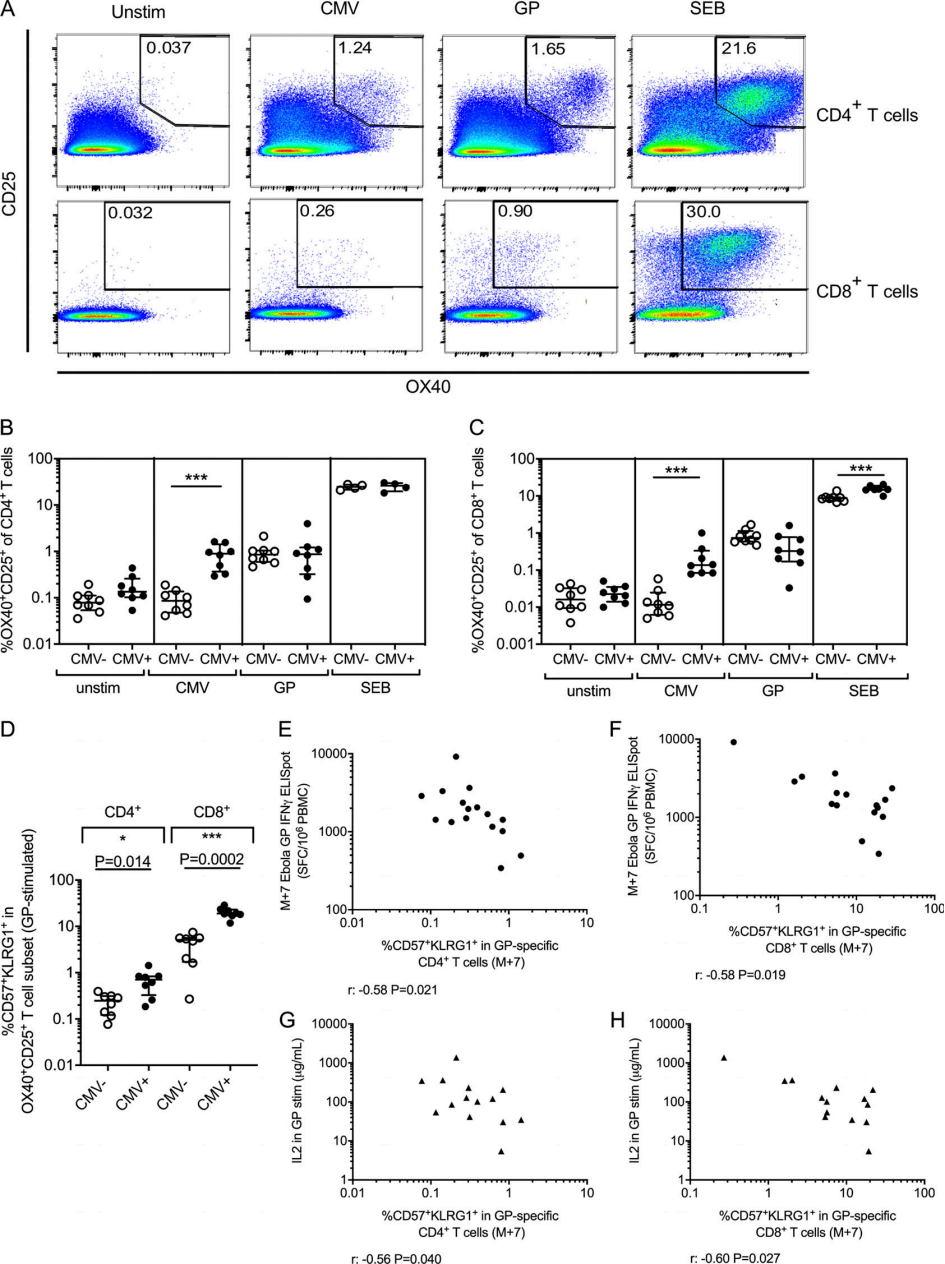
**Increased frequencies of antigen-specific T cells express CD57 and KLRG1 in CMV^+^ young adults.** **(A)** Expression of AIMs OX40 and CD25 on CD4^+^ and CD8^+^ T cells in PBMCs that were unstimulated (unstim) or stimulated with CMV pp65, Ebola GP, or Staphylococcal enterotoxin B (SEB). **(B and C)** Frequency of OX40^+^CD25^+^ cells in CD4^+^ T cells (B) and CD8^+^ T cells (C) in CMV^−^ (*n* = 8) and CMV**^+^** (*n* = 8) individuals. Mann–Whitney analyses. **(D)** Frequency of CD57^+^KLRG1^+^ cells within GP-specific (OX40^+^CD25^+^) CD4^+^ and CD8^+^ T cells in CMV^−^ (*n* = 8) and CMV**^+^** (*n* = 8) UK adults at M+7. Mann–Whitney analyses between CMV^−^ and CMV^+^ groups. **(E–H)** Relationship between frequency of CD57^+^KLRG1^+^ cells in CD4^+^ (E and G) or CD8^+^ (F and H) T cells and M+7 Ebola GP IFN-γ ELISPOT (E and F; CMV^−^ *n* = 8, CMV**^+^** *n* = 8) or IL2 produced by GP-stimulated (stim) M+7 PBMCs (G and H; measured using the LEGENDplex assay; CMV^−^, *n* = 8, and CMV**^+^**, *n* = 6; cytokine data for two individuals not available). Spearman’s rank analyses. SFC, spot-forming cell. Medians shown for all column graphs. Error bars indicate IQRs. *, P < 0.05; ***, P < 0.001.

Analysis of antigen-specific T cell phenotype and function (by antigen-stimulated cytokine production or using peptide-MHC class I and II tetramers) is difficult in clinical trials due to the wide range of MHC haplotypes, specificity for different peptides and heterogeneous cytokine production by different T cell subsets. Therefore, an alternative assay measuring activation-induced markers (AIMs), was used to determine the frequency and phenotype of antigen-responsive cells samples from the UK cohort (Fig. 4 A), as demonstrated previously (Dan et al., 2016; Havenar-Daughton et al., 2016; Bowyer et al., 2018).

The proportion of CD4^+^ and CD8^+^ T cells expressing OX40 and CD25 in response to stimulation with vaccine antigen (Ebola GP) or CMV (pp65) was assessed (Fig. 4, B and C). Background levels of expression in unstimulated cells were low (median 0.123%; interquartile range [IQR], 0.069%–0.313% for CD4^+^ and 0.031%; IQR, 0.017%–0.044% for CD8^+^ T cells). Significant CMV-specific CD4^+^ and CD8^+^ T cell responses were detected in CMV^+^ participants compared with CMV^−^ participants, in whom the proportion of AIM^+^ cells in the CMV pp65 stimulation was comparable to background. The frequency of Ebola GP–specific CD4^+^ and CD8^+^ T cells 7 d after MVA boost was comparable in CMV^+^ and CMV^−^ individuals, although some CMV^+^ individuals had lower GP-specific CD8^+^ T cell responses (CD4^+^: median 0.853% for CMV^−^ and 0.870% for CMV^+^; CD8^+^: median 0.731% for CMV^−^ and 0.329% for CMV^+^).

However, while the vaccine-specific T cell responses did not differ quantitatively between CMV^−^ and CMV^+^ individuals, there was a marked qualitative difference. In CMV^+^ individuals, both the Ebola GP–specific CD4^+^ and CD8^+^ T cells contained significantly higher proportions of CD57^+^KLRG1^+^ cells than in CMV^−^ participants. This was particularly pronounced in the CD8^+^ subsets, in which 19% were CD57^+^KLRG1^+^ in CMV^+^ participants compared with 5% in CMV^−^ participants (Fig. 4 D; P = 0.029 for CD4^+^ and P = 0.0003 for CD8^+^ T cells). Additionally, the proportions of CD57^+^KLRG1^+^ cells within both the Ebola GP–specific CD4^+^ and CD8^+^ T cells were negatively associated with IFN-γ and IL2 responses to GP (Fig. 4, E–H). Although CMV-associated changes in the global T cell repertoire have previously been observed, this is the first study to demonstrate phenotypic differences in antigen-responsive T cells after vaccination in CMV^+^ individuals.

### Pathogen exposure

**Figure 5.**
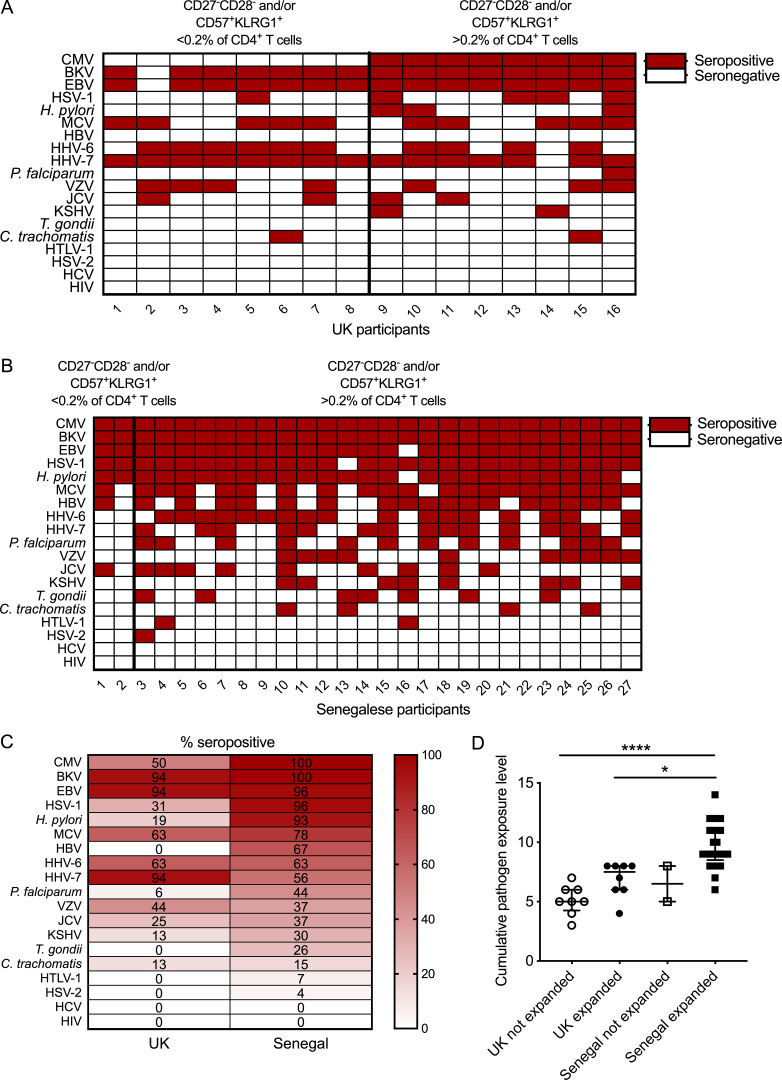
**Pathogen exposure.** Serostatus of each individual for 19 different pathogens. CMV and malaria serostatus determined by ELISA. Serostatus for all other pathogens was determined by multiplex serology. Individuals from each cohort are grouped according to whether an expanded population of CD57^+^KLRG1^+^ and/or CD27^−^CD28^−^ CD4^+^ T cells was present. **(A and B)** UK participants (A; *n* = 16) and Senegalese participants (B; *n* = 27), red = seropositive, white = seronegative. **(C)** Pathogen-specific seroprevalence in each cohort (UK, *n* = 16 and Senegal, *n* = 27). **(D)** Total number of seropositive results for each individual. Kruskal–Wallis analysis with Dunn’s post-test comparisons (adjusted P values: Senegalese volunteers with expanded CD57^+^KLRG1^+^/CD27^−^CD28^−^ CD4^+^ T cells versus UK volunteers without expanded CD57^+^KLRG1^+^/CD27^−^CD28^−^ CD4^+^ T cells, P < 0.0001; Senegalese volunteers with expanded CD57^+^KLRG1^+^/CD27^−^CD28^−^ CD4^+^ T cells versus UK volunteers without, P = 0.016). Medians shown and error bars indicate IQRs. BKV, BK virus; HBV, hepatitis B virus; HCV, hepatitis C virus; HHV-6, human herpesvirus 6; HHV-7, human herpesvirus 7; HSV-1, HSV type 1; HSV-2, HSV type 2; HTLV-1, HTLV type 1; JCV, JC virus; KSHV, Kaposi’s sarcoma-associated herpesvirus; MCV, Merkel cell polyomavirus; VZV, varicella zoster virus. *, P < 0.05, ****, P < 0.0001.

A number of other chronic or repeated infections have previously been shown to influence immune phenotypes and have an impact on vaccine responses (Stelekati and Wherry, 2012). All participants were negative for acute or chronic hepatitis B, hepatitis C, HIV, and *Plasmodium spp.* infections at enrollment. Serostatus for 19 different pathogens was determined for all participants in both cohorts (Fig. 5)

In the UK cohort, only CMV^+^ individuals had populations of CD57^+^KLRG1^+^ and/or CD27^−^CD28^−^ cells that made up >0.2% of the total CD4^+^ T cell compartment (Fig. 5 A). No other pathogen was exclusive to either group within this cohort.

In addition to CMV, almost all Senegalese individuals were seropositive for HSV-1 and *Helicobacter pylori*, and around half had evidence of significant exposure to *Plasmodium falciparum* (Fig. 5, B and C). These and other pathogens such as helminths have been associated with reduced vaccine responses or immune suppression and could play a role in the reduced immunogenicity observed in this population (Williamson and Greenwood, 1978; Sabin et al., 1996; Cooper et al., 2001; Muhsen et al., 2014). Many of these pathogens also influence the course of infection and development of immune responses against other pathogens, including causing the reactivation of latent viruses such as CMV (Stelekati and Wherry 2012; Stowe et al., 2012; Ogunjimi et al., 2014). However, in our study it was CMV that was clearly associated with the expansion of terminally differentiated CD4^+^ T cells and a reduction in vaccine responses.

The “cumulative pathogen exposure level” based on the number of seropositive results was increased in UK CMV^+^ (median, 7.5) and Senegalese (median, 9) participants compared with UK CMV^−^ participants (median, 5; Fig. 5 D). The two Senegalese volunteers with low frequencies of CD27^−^CD28^−^ and CD57^+^KLRG1^+^ CD4^+^ T cells both had relatively low cumulative pathogen exposure levels (5 and 8) and relatively low titers of CMV IgG (18 and 21 standard units compared with the median of 26 standard units in the Senegalese cohort).

The nature of Phase I vaccine trials means that the sample size in each population was relatively small and was not powered for multivariate analyses. In future trials involving larger numbers of individuals, it would be of clear value to conduct a similar analysis and determine the individual and combined effects of different chronic pathogens on vaccine responses.

### Concluding remarks

These Ebola vaccine trials, run concurrently in healthy young UK and Senegalese adults, allowed for a direct comparison of vaccine immunogenicity in a developed country and a developing country. The results of this study suggest that high CMV seroprevalence may have a role in driving the reduced vaccine immunogenicity observed in some developing countries. This has important implications for future vaccine studies, particularly when comparing trial outcomes between populations with different CMV seropositivity rates. As is evident by recent epidemics of novel pathogens, including Ebola, it is of clear importance that young and older adults are able to mount an effective response to novel antigens. Therefore, our finding that CMV carriage was associated with a reduction in the response to a novel antigen in young adults implies that CMV might have a broader impact on public health than previously expected.

## Materials and methods

### Study populations

Cryopreserved peripheral blood mononuclear cells (PBMCs) and plasma from two Phase I clinical trials were used in this study. The UK cohort consisted of the 16 volunteers in group 2 from EBL04 (ClinicalTrials.gov registration ref: NCT02451891), and the Senegalese cohort consisted of all 40 volunteers in EBL06 (NCT02485912). The UK study was conducted in healthy adults aged 18–50 yr (average age, 33 yr) at the Centre for Clinical Vaccinology and Tropical Medicine, University of Oxford, UK. The Senegalese study was conducted in healthy adults aged 18–50 yr (average age, 28 yr) at the Centre Hospitalier Universitaire le Dantec, Dakar, Senegal. All volunteers received 3.6 × 10^10^ viral particles of ChAd3–EBO-Z followed by 1.0 × 10^8^ plaque-forming units of MVA–EBO-Z 7 d later. Both vaccinations were delivered intramuscularly into the deltoid region of the arm. Further details of both studies can be found in the clinical trial paper (Venkatraman et al., 2018) and study protocols, which were submitted with the clinical trial manuscript.

### CMV seroprevalence

CMV seroprevalence was assessed in baseline plasma samples by a commercially available ELISA kit (Abcam; ab108724) according to the manufacturer’s instructions.

### Vaccine-induced antibody responses

Antibody responses to vaccination were assessed using a standardized ELISA for total IgG against trimeric Zaire Ebola GP as previously described (Venkatraman et al., 2018). A reference pool of positive serum was used to form a standard curve. Arbitrary ELISA units were calculated for each sample using the OD values of the sample and the parameters of the standard curve. All ELISAs were conducted by the same operator at the Jenner Institute, University of Oxford.

### Vaccine-induced T cell responses

T cell responses against Zaire Ebola GP were assessed using ex vivo (18-h stimulation) IFN-γ ELISPOT assays as previously described (Venkatraman et al., 2018). Assays were conducted using fresh PBMCs, therefore performed in Oxford for the UK cohort and in Dakar for the Senegalese cohort. The same protocol was used for both cohorts, and a thorough process of technology transfer and training was conducted before study commencement to minimize assay variation between the trial sites.

### T cell phenotyping

T cell phenotyping was conducted in Oxford for all 16 UK volunteers and 27 Senegalese volunteers for whom there were cryopreserved cells available. PBMCs were thawed and rested for 2 h at 37°C in 2.5 µl/ml Benzonase Endonuclease (E1014-25KU; Sigma-Aldrich). One to two million PBMCs for each individual were stained in 50 µl in 96-well plates. Cells were incubated for 20 min at room temperature (RT) with the Live/Dead Aqua Dead Cell Stain Kit (Invitrogen) then surface stained at RT with CD14-eF506 (61D3, 1/50; eBioscience), CD19-eF506 (HIB19, 1/50; eBioscience), CD45RA-eV605 (HI100, 1/50; eBioscience), CD27-BV711 (O323, 3/50; BioLegend), CD28-BV421 (CD28.2, 1/50; BioLegend), CD4-APC (RPA-T4, 1/50; eBioscience), CD3-AF700 (UCHT1, 1/50; eBioscience), CD8-APC-AF780 (RPA-T8, 3/50; eBioscience), CCR7-FITC (G043H7, 1/50; BioLegend), CD57-PerCP-Cy5.5 (HNK-1, 1/50; BioLegend), and KLRG1-PE (14C2A07, 3/50; BioLegend). Cells were acquired immediately using a BD LSRII. Data analysis was conducted in FlowJo version 10.1 (Treestar Inc). Gating strategy is shown in Fig. S1.

### AIM assay

The AIM assay was conducted in a similar way to previously published work (Dan et al., 2016; Havenar-Daughton et al., 2016; Bowyer et al., 2018). PBMCs were thawed as for the T cell phenotyping assay. PBMCs were stimulated overnight (20 h at 37°C) with 1–2 × 10^6^ cells per well in a 96-well U-bottom plate. Cells were either stimulated with 0.5 µg/ml of a pool of CMV pp65 peptides (PM-PP65-1; Cambridge Bioscience) or 2 µg/ml of Zaire Ebola GP peptides (Neoscientific). An unstimulated well and a positive control well stimulated with 1 µg/ml staphylococcal enterotoxin B were included for each sample. After overnight stimulation, cells were washed twice in FACS buffer (PBS with 1% BSA and 0.1% NaN_3_) and then stained for 20 min at RT with 100 µl per well of the following fluorescently conjugated antibodies: αCD45RA-eV605 (HI100, 1/200; eBioscience), αCD14-eF506 (61D3, 1/200; eBioscience), αCD19-eF506 (HIB19, 1/200; eBioscience), αCD4-APC-eF780 (RPA-T4, 1/100; eBioscience), αCD3-AF700 (UCHT1, 1/133; eBioscience), αOX40-PE/Cy7 (Ber-ACT35, 1/100; Biolegend), αCD25-FITC (M-A251, 1/100; BioLegend), αCD57-PE (HNK-1, 1/200; BioLegend), αKLRG1-BV421 (14C2A07, 1/66; BioLegend), αCD8-BV711 (RPA-T8, 1/400; BioLegend), and Aqua Live/Dead stain (1/2000). Cells were washed twice as before, fixed with 4% paraformaldehyde for 10 min at 4°C, washed twice again, resuspended in 100 µl FACS buffer, and acquired immediately on a BD LSRII. Data analysis was conducted in FlowJo version 10.

### Anti-schizont ELISA

*P. falciparum*–specific IgG was detected by anti-schizont ELISA conducted as previously described (Hodgson et al., 2015). A positive cutoff value of 0.25 OD405 was calculated based on the mean +3 standard deviations of 30 UK malaria-naive samples.

### Multiplex serology

Multiplex serology is a fluorescent bead-based high-throughput method allowing the simultaneous measurement of serum antibodies against a variety of pathogen-specific antigens (Waterboer et al., 2005, 2006). Serum antibodies against human herpesviruses 1–8, hepatitis B and C viruses, *H. pylori, Chlamydia trachomatis, Toxoplasma gondii*, human polyomaviruses BK, JC, and MC, HIV 1, and human T cell lymphotropic virus 1 were measured in the UK and Senegalese cohorts as previously described (Waterboer et al., 2005, 2006; Kjaerheim et al., 2007; Michel et al., 2009; Dondog et al., 2015; Brenner et al., 2018, 2019; Hulstein et al., 2018; Kranz et al., 2019). In brief, pathogen-specific antigens were recombinantly expressed as glutathione-S-transferase fusion proteins in *Escherichia coli* and in situ purified on fluorescent beads. Individual bead sets are differentially colored and distinguishable using a Luminex 200 flow cytometer. Each antigen was loaded onto one glutathione casein–coated bead set. In addition, glutathione-S-transferase was loaded onto one bead set for background subtraction. Antigen-loaded bead sets were combined into one bead mix and incubated with serum (final serum dilution, 1:1,000). Immunocomplexes consisting of primary serum antibodies bound to a pathogen-specific antigen were detected using a biotinylated IgG/IgM/IgA secondary antibody and streptavidin-R-phycoerythrin as a reporter dye. Antibody reactivities were quantified using a Luminex 200 flow cytometer as median fluorescence intensities from at least 100 beads per bead set and serum.

### Statistical analysis

Data are presented as medians and IQRs. Mann–Whitney analysis was used to compare CMV^−^ and CMV^+^ groups, and Kruskal–Wallis analysis with Dunn’s post-test was used for comparison across multiple groups. Spearman’s rank was used for linear regression analyses. An α-level of 0.05 was considered significant for all P values, and all tests were two-tailed. Analyses were performed in GraphPad Prism version 7.

### Study approval

Participants provided written informed consent before inclusion in these studies. Both studies were conducted according to the principles of the Declaration of Helsinki (2008) and the International Conference on Harmonization Good Clinical Practice guidelines. The UK study protocol and associated documents were reviewed and approved by the UK National Research Ethics Service (Committee South Central–Oxford A, Ref: 15/SC/0108), the Medicines and Healthcare Products Regulatory Agency (Ref: 21584/0341/001-0001), and the Oxford University Clinical Trials and Research Governance team, who independently and externally monitored compliance with Good Clinical Practice guidelines. Vaccine use was authorized by the Genetically Modified Organisms Safety Committee of the Oxford University Hospitals NHS Trust (Ref: GM462.15.82). An independent local safety monitor provided safety oversight. The trial was registered with ClinicalTrials.gov (Ref: NCT02451891). Ethical approval for the Senegalese study was granted in the UK by the Oxford Tropical Research Ethics Committee (Ref: 27–15). Ethical and regulatory approvals for this study were also granted in Senegal by the Senegal Comité National d’Ethique pour la Recherche en Santé and the Senegalese Regulatory authority, the Ministry of Health, and the Social Action Department of Pharmacy and Laboratories.

### Online supplemental material

Fig. S1 shows the gating strategy for T cell memory phenotyping, and Fig. S2 details the differences in CD4^+^ and CD8^+^ memory T cell populations between CMV^−^ and CMV^+^ individuals. Table S1 summarizes the demographics of each cohort.

## Supplementary Material

Table S1 presents the demographics and baseline characteristics of the Senegalese and UK participants.
