# Supplementary material for: Reduced Ebola vaccine responses in CMV+ young adults is associated with expansion of CD57+KLRG1+ T cells
Source: J Exp Med. 2020 May 15;217(7):e20200004. doi: 10.1084/jem.20200004 (PMC7336307; doi:10.1084/jem.20200004)
Supplement: Table S1 — presents the demographics and baseline characteristics of the Senegalese and UK participants. [file JEM_20200004_TableS1.docx]

Table S1. Demographics and baseline characteristics of Senegalese, UK CMV^−^, and UK CMV^+^ volunteers

| Cohort | | UK CMV^−^ | UK CMV^+^ | Senegal |
| --- | --- | --- | --- | --- |
| Number of volunteers, *n* | | 8 | 8 | 40 |
| Gender, *n* (%) | Female | 6 (75) | 4 (50) | 11 (27.5) |
|  | Male | 2 (25) | 4 (50) | 29 (72.5) |
| Age, yr | 18–20 |  |  | 9 (22.5) |
|  | 21–30 | 5 (62.5) | 4 (50) | 17 (42.5) |
|  | 31–40 | 1 (12.5) | 2 (25) | 12 (30) |
|  | 41–50 | 2 (25) | 2 (25) | 2 (5) |
|  | Mean | 33 | 33 | 28 |
| Ethnicity^a^ | White | 7 (87.5) | 6 (75) | 0 |
|  | Black | 0 | 1 (12.5) | 40 (100) |
|  | Asian | 1 (12.5) | 0 | 0 |
|  | Mixed | 0 | 1 (12.5) | 0 |
|  | Other | 0 | 0 | 0 |
| Body mass index^b^ | <18.5 |  |  | 1 (2.5) |
|  | 18.5–24.9 | 3 (37.5) | 3 (37.5) | 27 (67.5) |
|  | 25–29.9 | 2 (25) | 3 (37.5) | 7 (17.5) |
|  | ≥30 | 3 (37.5) | 2 (25) | 4 (10) |
|  | Mean | 28 | 27 | 23.7 |

All data are presented as *n* (%) unless otherwise noted.

^a^Ethnicity was self-reported.

^b^Body mass index is the weight in kilograms divided by the square of the height in meters. Height was not recorded in one Senegalese volunteer. All Senegalese participants were CMV^+^.
